# G3BP1 coordinates lysophagy activity to protect against compression‐induced cell ferroptosis during intervertebral disc degeneration

**DOI:** 10.1111/cpr.13368

**Published:** 2022-11-30

**Authors:** Shuai Li, Zhiwei Liao, Huipeng Yin, Ouyang Liu, Wenbin Hua, Xinghuo Wu, Yukun Zhang, Yong Gao, Cao Yang

**Affiliations:** ^1^ Department of Orthopaedics, Union Hospital, Tongji Medical College Huazhong University of Science and Technology Wuhan China

## Abstract

Lysophagy is a form of selective autophagy to remove unwanted lysosomes. However, its role in the pathogenesis of intervertebral disc degeneration (IDD) remains unclear. We intended to investigate the relationship between lysophagy and ferroptosis, as well as the potential involved molecules during IDD. Human nucleus pulposus (NP) cells were obtained from clinical patients. The protein levels, protein colocalization and cellular reactive oxygen species levels were assessed by western blotting, immunofluorescence analysis, immunoprecipitation and flow cytometry, respectively. The in vivo experiments were conducted based on the needle puncture‐induced IDD model in rats. Compression pressure induces the lysophagy inactivation and lysosomal damage, resulting in iron overload and ferroptosis in human NP cells. Notably, Ras GTPase‐activating protein‐binding proteins 1 (G3BP1) resides at lysosomes to coordinate lysophagy activity mainly via the function of G3BP1/TSC2 complex. Dysfunction of G3BP1/TSC2 complex accelerates the lysosomal damage and ferroptosis in NP cells. Besides, inhibition of mTOR signalling ameliorates lysosomal damage and protects against cell ferroptosis. The in vivo experiments also demonstrate that the G3BP1/mTOR signalling is involved in the progression of IDD. These findings illustrate the relationship between lysophagy and compression‐induced cell ferroptosis. It also indicates the positive role of G3BP1 and may provide potential targets for IDD treatment.

## Graphical abstract

G3BP1 coordinates TSC/mTOR complex to initiate lysophagy for removing injured lysosomes. Under the compression pressure, G3BP1 dysfunction promotes the inactivation of lysophagy. Accumulation of damaged lysosomes induces the elevated levels of free iron and lipid peroxidation, resulting in cell ferroptosis.

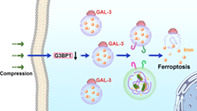

## 1 INTRODUCTION

Low back pain associated with degenerative disc disease (DDD) is a leading cause of disability that brings a huge medical burden worldwide.^1^ Intervertebral disc degeneration (IDD) is the main aetiology of DDD, including the pathophysiological changes of disc biology and biomechanics.^2^ Intervertebral disc (IVD) is composed of the inner gelatinous nucleus pulposus (NP), the surrounding annulus fibrosus (AF) and the cartilaginous endplates at both ends.^3^ During the progression of IDD, the NP tissues are diminished increasingly and replaced by fibrotic and calcified tissues due to the degradation of extracellular matrix (ECM), loss of resident cells and the release of inflammatory cytokines.^3^, ^4^ Currently, probably erroneously, most approached for treating IDD have been concentrated on the repair of NP tissues and the NP cells.^5^ How to enhance NP cell viability and reduce mortality becomes an urgent problem in developing IDD treatments. Therefore, it is necessary to understand the biological basis of NP cells and investigate the pathological mechanisms during the progression of IDD.

Lysosomes, the major degradative organelles, are essential for the maintenance of cellular homeostasis.^6^ Lysosomal damage induced by various endogenous or exogenous factors can directly injure cells via the release of cathepsins and hydrolases, or trigger other cell death pathways.^7^ In response to lysosomal damage, lysophagy as an important endogenous protective mechanism plays a role in preventing irreversible cell damage. Lysophagy is the process that refers to the clearance of damaged lysosomes by selective autophagy.^8^ During the lysophagy, galectins, especially galectin‐3, sense the damaged lysosomes and recruit autophagy regulatory molecules, such as E3 ubiquitin ligase‐tripartite domain containing protein 16 (Trim16), resulting in the formation of autophagosome and the subsequent degradation of lysosome.^9^ Importantly, the AAA‐ATPase VCP/p97 plays a critical role in driving lysophagy, which cooperates other molecules to target ubiquitinated substrate proteins for degradation.^8^, ^10^ Therefore, lysophagy is required for sustaining lysosome function and lysosome damage is involved in the development of various diseases.^9^ Despite previous studies have investigated the role of lysosome‐related autophagy in degenerative NP cells,^11^, ^12^, ^13^ the specific function of lysophagy during the progression of IDD still remains unknown.

Lysosomal damage is associated with many types of regulated cell death pathways, including ferroptosis.^9^ Ferroptosis mainly caused by reactive oxygen species (ROS) is a type of cell death which is related to iron‐dependent lipid hydroperoxides.^14^, ^15^ Since lysosome is a main organelle with iron storage,^16^ ferroptosis may be closely linked with lysosomal damage or lysophagy. Upon severe lysosomal damage, the free iron can be released from damaged lysosomes and elicit the ROS production, leading to cell ferroptosis.^17^ Clearance of lysosome could enhance the release of iron via the autophagic degradation of ferritin.^16^ Collectively, lysosomes regulate ferroptosis through altering the levels of iron‐uptake and iron‐efflux. The regulatory mechanisms still remain unknown when it comes to damaged lysosomes and lysophagy activity. Previous studies have indicated the existence of ferroptosis in NP cells and tentatively explored its role during IDD.^18^, ^19^, ^20^ However, the underlying mechanisms involved in ferroptosis‐related disc degeneration are complicated and need to be further investigated.

Ras GTPase‐activating protein‐binding proteins 1 (G3BP1) is a well‐known component of stress granules,^21^ but it is far beyond that and has diverse function. Growing evidence suggests the role of G3BP1 in regulating cell senescence, immune response and apoptosis.^22^, ^23^, ^24^ A recent study has indicated that G3BP1 could modulate the activation of the mechanistic target of rapamycin (mTOR) signalling pathway, acting as the role of a lysosomal tether.^25^ These discoveries supported that G3BP1 could influence the lysosomal function, probably including the quantity and activity of lysosomes. Therefore, it is reasonable to suspect that G3BP1 plays a role in regulating lysophagy activity and lysosomal repair under stress condition. Besides, the relationship between G3BP1 and mTOR signalling pathway still needs to be verified in NP cells during the progression of IDD.

In this study, we speculated that G3BP1 mediated lysophagy and lysosome activity via regulating cell ferroptosis during the IDD process. We measured the expression levels of G3BP1 and the lysosomal damage marker Galectin‐3 in human NP tissues, and found the correlation between their expression and degenerative degree of discs. In the in vitro compression model, lysophagy played a role in NP cell ferroptosis and G3BP1 acted as a lysosomal modulator via the interaction of mTOR signalling. The roles of G3BP1 and mTOR signalling were further investigated in the in vivo disc degeneration model. Therefore, this study deepens the understanding of the relationship between G3BP1, lysophagy and ferroptosis in NP cells, and provides new targets for IDD therapy.

## 2 MATERIALS AND METHODS

### 2.1 Tissues collection and classification

The degenerative degree of human NP tissues was classified according to the Pfirrmann grading scale based on the magnetic resonance images.^26^ Discs categorized as Pfirrmann grade I or II was considered as non‐degenerative (NC) group, and are mainly from patients diagnosed as lumbar fracture or idiopathic scoliosis. Other NP tissues collected from patients who underwent disc fusion surgery due to DDDs were classified as degenerative (IDD) group, and these discs are mainly categorized as Pfirrmann grade III, IV, or V. Human NP tissues were obtained and fixed in 4% formaldehyde, and then embedded in paraffin for the histological analysis. Some samples were directly used for cell isolation or frozen in liquid nitrogen for storage. Fully informed consent was obtained from all patients, and all experimental protocols were approved by the Ethics Committee of Tongji Medical College, Huazhong University of Science and Technology.

### 2.2 Cell culture and compression treatment

Human NP cells were isolated as previously described.^27^ Briefly, NP tissues were collected and cut into pieces in a sterile tube. Then, these tissues were enzymatically digested in 0.2% type II collagenase for 4 h. After washed with phosphate‐buffered saline (PBS) twice and centrifuged, the sediments were resuspended with Dulbecco's Modified Eagle Medium/Nutrient Mixture F‐12 (DMEM/F‐12) containing 15% fetal bovine serum (HYCEZMBIO) in a 5% CO_2_ incubator at 37°C. The culture medium was replaced once every 3 days. In the static pressure model, the NP cells were cultured in plates and placed in a compression apparatus under humidified atmosphere at 37°C as previously described.^28^ The mixed air (0.5% CO_2_ and 99.5% compressed air) was pumped into the apparatus and it could achieve a 1.0 MPa static pressure.

### 2.3 Western blot

For protein extraction, cells were treated with RIPA buffer with a protease inhibitor PMSF (Beyotime). The isolated proteins were separated by sodium dodecyl sulphate‐polyacrylamide gel electrophoresis (SDS‐PAGE), and then transferred onto a PVDF membrane. The bands were blocked with 5% skim milk and then incubated with a primary antibody overnight. The primary antibodies used were as follows: G3BP1 (Abcam, ab181150), Galectin‐3 (CST, 87985 S), FTL (Proteintech, 10727‐1‐AP), GPX4 (Proteintech, 14432‐1‐AP), Trim16 (Proteintech, 24403‐1‐AP), VCP/p97 (Proteintech, 10736‐1‐AP), mTOR (Proteintech, 66888‐1‐Ig), p‐mTOR (Proteintech, 67778‐1‐Ig), EIF3A (Proteintech, 67713‐1‐Ig), EIF2S1 (Proteintech, 28740‐1‐AP) and GAPDH (Proteintech, 60004‐1‐Ig). After washed with washing buffer, the bands were incubated with a horseradish peroxidase‐conjugated secondary antibody (Proteintech) for 1 h, and then visualized using the chemiluminescence system. The band intensity was measured by ImageJ 1.8 software (National Institutes of Health).

### 2.4 Quantitative real‐time polymerase chain reaction

NP cells were treated with TRIzol (Invitrogen) RNA isolation reagent, and RNA was separated by chloroform. Then, RNA was reverse‐transcribed and amplified by quantitative real‐time polymerase chain reaction (qRT‐PCR) according to the previous protocol.^26^ The primers used were as follows: Homo G3BP1, forward 5′‐AGGTGAGGTCCGTCTGAATG‐3′, reverse 5′‐CCCTTCCCACTCCAAATCCT‐3′; Homo Galectin‐3, forward 5′‐AGGGAAGAAAGACAGTCGGT‐3′, reverse 5′‐ATGAAGCACTGGTGAGGTCT‐3′; Homo TSC2, forward 5′‐AAAACCAAACAGCGCGAGAT‐3′, reverse 5′‐GCGGCAAAGTTCCTGTAGAG‐3′; Homo GAPDH, forward 5′‐TCAAGAAGGTGGTGAAGCAGG‐3′, reverse 5′‐TCAAAGGTGGAGGAGTGGGT‐3′. GAPDH as an internal control was used for normalization. All experiments were conducted in triplicate.

### 2.5 Assessment of iron levels

Cellular iron levels were measured using an iron assay kit (Abcam) according to the manufacturer's instructions. Briefly, samples were homogenized in 5× volume of iron assay buffer on ice, and the supernatant was obtained by centrifugation (16,000*g*, 10 min). Then 5 μl iron reducer was added into the samples for incubation (30 min, 37°C), and subsequently 100 μl of iron probe was added for incubation (60 min, 37°C) in the dark. Optical density was immediately measured at 593 nm absorbance using a spectrophotometer (BioTek).

### 2.6 Measurement of lipid ROS levels

Lipid ROS levels were measured using a lipid peroxidation sensor (C11‐BODIPY 581/591, Thermo Fisher Scientific) according to the manufacturer's protocol. Briefly, cells were incubated with 10 μM C11‐BODIPY 581/591 at 37°C for 30 min. Then, cells were washed with PBS gently and placed under the microscope (Olympus, BX53). The fluorescent images at green and red fluorescent channels were obtained by three independent researchers, respectively. The mean fluorescence intensity of images was analysed by ImageJ 1.8 (National Institutes of Health).

### 2.7 Propidium iodide staining

Cell death was evaluated by propidium iodide (PI) staining. NP cells were cultured in a six‐well plate and treated with interests for scheduled time. Then NP cells were stained with PI (50 μg/ml) for 30 min. The nuclei were stained by DAPI (0.1 g/ml, 5 min). The fluorescent images were obtained under a microscope (Olympus, BX53) by three independent researchers. The PI‐positive cells were calculated by ImageJ 1.8 (National Institutes of Health).

### 2.8 Immunofluorescence staining

NP cells cultured in six‐well plates were fixed with 4% paraformaldehyde for 30 min, and then permeabilized with Triton X‐100 (0.2%, 30 min). The samples were washed in PBS twice, and blocked with 2% goat serum for 1 h. Then the samples were incubated with primary antibodies overnight, and subsequently incubated with FITC or Cy3‐conjugated secondary antibody. After the nuclei were stained for DAPI (0.1 g/ml, 5 min), the fluorescent images were captured using a fluorescence microscope (Olympus, BX53) or a confocal microscope (Nikon A1R SI Confocal, Japan).

### 2.9 RNA interference

RNA interference of target genes in NP cells was realized by small interfering RNA (siRNA) transfection. Target siRNA and scrambled siRNA (si‐scr) were synthesized by RiboBio company (Guangzhou, China), and the transfection was conducted according to the manufacturer's protocol. The sense sequences of siRNAs used were listed as follows: G3BP1‐siRNA1, 5′‐GGGCUUCUCUCUAACAACATT‐3′; G3BP1‐siRNA2, 5′‐GCGAGAACAACGAAUAAAUTT‐3′; G3BP1‐siRNA3, 5′‐CCGACAAAUCAGAGCUUAAATT‐3′; TSG2‐siRNA1, 5′‐GCAUGGAAUGUGGCCUCAATT‐3′; TSG2‐siRNA2, 5′‐GGGACAUUCUGCUGAACAUTT‐3′; TSG2‐siRNA3, 5′‐GCCACACACACCACUUCAATT‐3′ and negative control, 5′‐UUCUCCGAACGUGUCACGUTT‐3′. The transgenic efficacy was then detected using qRT‐PCR at 24 h after transfection.

### 2.10 Immunoprecipitation

NP cells were treated with a mixed buffer (50 mM Tris–HCl, 150 mM NaCl, 1 mM EDTA and 1% NP‐40) with protease inhibitor cocktail. The sample was centrifuged (12,000*g*, 30 min) and the supernatant was collected. Then the sample (500 μg) was added with 10 μl of the primary antibodies for immunoprecipitation, and was incubated overnight at 4°C with magnetic beads (MCE). IgG was used as a negative control. The immunoprecipitates were separated by magnetic force, and then conducted with Western blot assays.

### 2.11 Animal experiments

All protocols involved in animal experiments were approved by the Animal Experimentation Committee of Huazhong University of Science and Technology. Sprague–Dawley (SD, male, 2 months, 200 g) rats were purchased from the Experimental Animal Center of Tongji Medical College, Huazhong University of Science and Technology. A surgical model of IDD was conducted by needle puncture as previously described.^26^ The discs of rat (Co 6/7, 7/8, 8/9 and 9/10) were marked by palpation and verified by radiography. The sham control group was punctured with the 33‐gauge needle and the IDD group was punctured with 20‐gauge. The 20‐gauge needle was used to initiate disc degeneration, while the 33‐gauge with less injury was used for the drug or target siRNA (2 μl in volume) injection.^29^ The injection procedure was repeated weekly for 2 months.

### 2.12 Radiography and magnetic resonance imaging

The rats were conducted with radiography using an in vivo MS FX PRO imaging system (Bruker). The disc height was measured using the ImageJ 1.8 software and calculated using the disc height index (DHI) according to a previously described.^30^ The change of DHI was used to evaluate disc degeneration and calculated according to the formula: DHI % = post‐DHI/pre‐DHI × 100%. Post‐DHI was the post‐operation DHI and pre‐DHI was the pre‐operation DHI. Besides, magnetic resonance imaging (MRI) was performed using an MRI system (BRUKER BioSpec), and sagittal T2‐weighted images were used to assess the signal of the discs, indicating the change of water content. Pfirrmann grades based on the T2‐weighted section images were used to evaluate the degree of IDD as previously described.^30^

### 2.13 Histological analysis

The rats were euthanized and the discs were harvested. The samples were fixed with 4% paraformaldehyde, and decalcified with EDTA, and then embedded in paraffin. For histological staining, the samples were cut into 4‐μm slices, and then deparaffinized, rehydrated, and stained with haematoxylin and eosin (HE) and safranin‐O (SO). The histological grades were evaluated as previously described.^18^ For immunochemistry staining, the slice of sample was deparaffinized, rehydrated and microwaved in sodium citrate. After blocked with bovine serum albumin for 30 min, the sections were incubated with primary antibodies overnight at 4°C. Finally, the sections were incubated with a secondary antibody and stained with haematoxylin.

### 2.14 Statistical analysis

Data were analysed using GraphPad Prism 8 software and presented as mean ± standard deviation (SD) of at least three independent experiments. Student's *t*‐test and one‐way or two‐way analysis of variance (ANOVA) with Tukey's post hoc test were used to compare data from different groups. Kruskal–Wallis *h*‐test was used to analyse the difference among multiple groups. The correlation was assessed by the non‐parametric linear regression. Values of *p* < 0.05 (*p < 0.05; **p < 0.01; ***p < 0.001) were with statistical significance, while *p* > 0.05 (*ns*) with no significant difference.

## 3 RESULTS

### 3.1 Decreased G3BP1 expression was detected in human degenerated NP tissues

**FIGURE 1:**
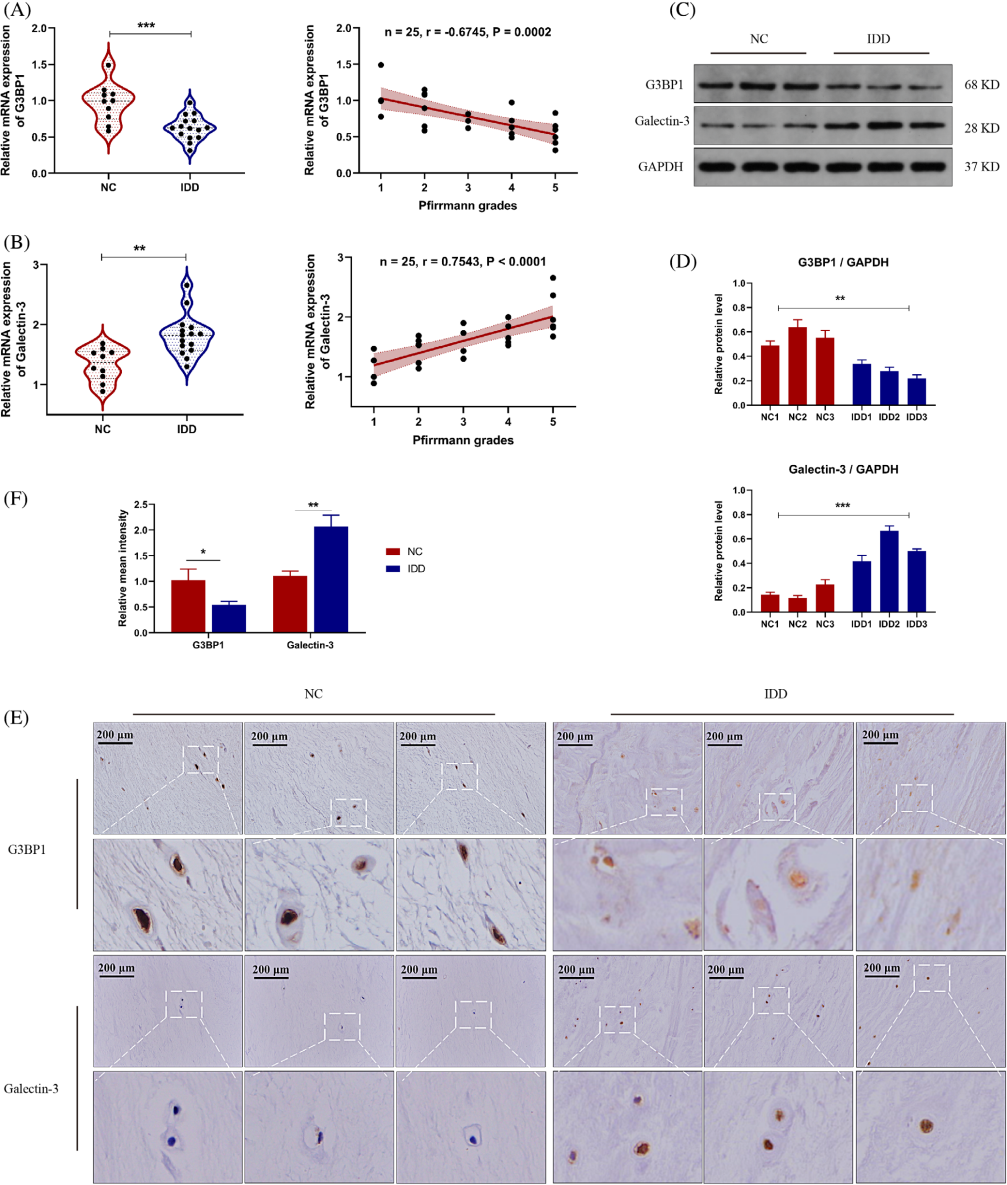
The expression of G3BP1 and Galectin‐3 in human NP tissues. (A) The relative mRNA level of G3BP1 in non‐degenerative (NC) and degenerative (IDD) human NP tissues (left). The regression analysis of G3BP1 mRNA level and Pfirrmann grade of human NP tissues (right). (B) The relative mRNA level of Galectin‐3 in non‐degenerative and degenerative human NP tissues (left). The regression analysis of Galectin‐3 mRNA level and Pfirrmann grade of human NP tissues (right). (C) Western blot analysis of G3BP1 and Galectin‐3 in non‐degenerative and degenerative human NP tissues. (D) Relative quantitative protein levels of G3BP1 and Galectin‐3. (E) The immunochemistry staining of G3BP1 and Galectin‐3 in non‐degenerative and degenerative human NP tissues. (F) Relative quantitative intensity of G3BP1 and Galectin‐3. IDD, intervertebral disc degeneration; NP, nucleus pulposus. Data were presented as mean ± SD (*n* = 3). *p < 0.05, **p < 0.01 and ***p < 0.001.

We first measured the expression levels of G3BP1 and Galectin‐3, a marker of lysosomal damage, in human NP tissues. The degenerative grade of NP tissues was evaluated based on the T2‐weighted MRI images of the donors. It was indicated that the transcription level of G3BP1 in the non‐degenerative group was higher than in the degenerative group (Figure 1A). We also found that the mRNA level of Galectin‐3 was increased in the degenerative NP tissues (Figure 1B). Both the levels of G3BP1 and Galectin‐3 were correlated with degenerative grade of NP tissues (Figure 1A,B). Meanwhile, according to the western blot analysis, it was shown that the protein level of G3BP1 was significantly decreased and Galectin‐3 was increased in degenerative NP tissues (Figure 1C,D). The immunochemistry results also found that a low expression level of G3BP1 and high expression level of Galectin‐3 in the degenerative NP tissues (Figure 1E,F). These results revealed that the level of G3BP1 decreases gradually as well as increased lysosomal damage during the progression of IDD.

### 3.2 Compression treatment induced the lysosomal damage and ferroptosis of NP cells in vitro

**FIGURE 2:**
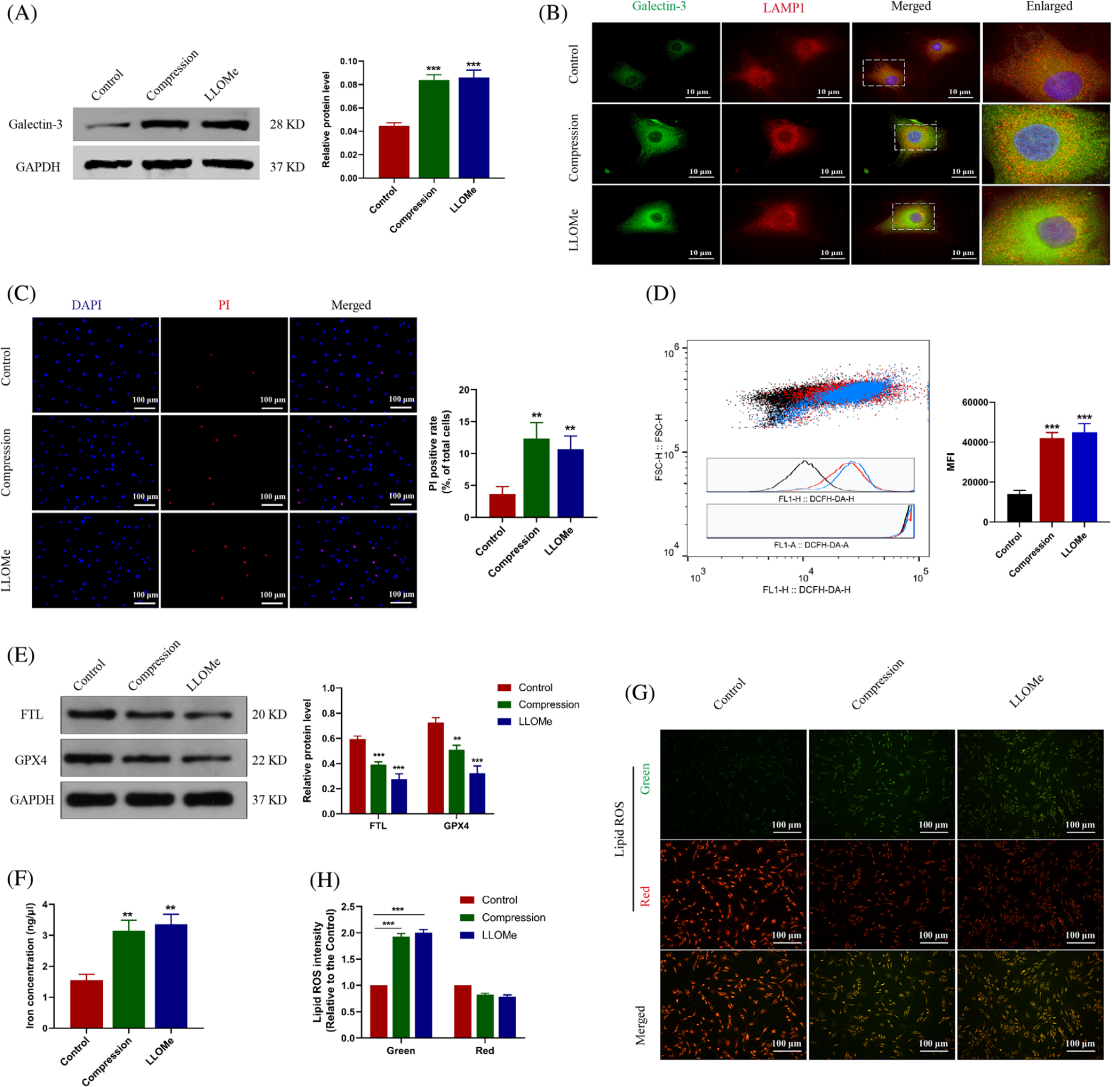
Compression treatment induced the lysosomal damage and ferroptosis of NP cells. (A) NP cells were treated with the compression pressure or LLOMe. Western blot analysis and quantitative protein levels of Galectin‐3 were shown. (B) Representative immunofluorescence images of Galectin‐3 (Green) and LAMP1 (red). (C) Representative PI staining images of NP cells and the quantitative PI‐positive cell rate. (D) Flow‐cytometry of DCFH‐DA indicating ROS level was shown and the mean fluorescence intensity (MFI) was calculated. (E) Western blot analysis and quantitative protein levels of FTL and GPX4 were shown. (F) The concentration of cellular free iron was measured. (G) Representative immunofluorescence images of C11‐BODIPY staining indicating the lipid ROS level. (F) Lipid ROS level was calculated based on the C11‐BODIPY staining results. FTL, ferritin light chain; NP, nucleus pulposus; PI, propidium iodide; ROS, reactive oxygen species. Data were presented as mean ± SD (*n* = 3). **p < 0.01 and ***p < 0.001.

Compression pressure is the common pathogenic factor of IDD.^31^ Herein, we utilized static compression treatment to simulate the external environment around NP cells. L‐leucyl‐L‐leucine methyl ester (LLOMe) was able to accumulate in lysosomes and was used to induce lysosomal damage. We observed that the protein level of Galectin‐3 was increased in both the compression and LLOMe group (Figure 2A). Immunofluorescence of Galectin‐3 and the lysosomal marker LAMP1 showed that the compression treatment induced the accumulation of Galectin‐3‐positive lysosome in NP cells (Figure 2B). It revealed the increased lysosomal damage in NP cells upon the compression pressure. On the other hand, we found that the compression treatment increased the death rate of NP cells (Figure 2C). To further investigate whether ferroptosis occurs, NP cells were incubated with DCFH‐DA probe. We found that the cellular ROS level was increased upon the compression treatment (Figure 2D). Some proteins, such as glutathione peroxidase 4 (GPX4) and ferritin light chain (FTL) are involved in regulating cell ferroptosis.^32^ We found that the protein levels of GPX4 and FTL were both decreased in the compression group (Figure 2E). It was revealed that lysosomal damage is companied with cell ferroptosis upon the compression pressure. Meanwhile, the concentration of cellular free iron was increased in the compression group (Figure 2F). It may be possible that free iron was released from the severely damaged lysosome. The lipid ROS level indicates the degree of lipid peroxidation, which is the hallmark of ferroptosis.^33^ It was indicated that both the compression and LLOMe treatment induce the increased level of lipid ROS (Figure 2G,H). These results indicated that the compression treatment induces the lysosomal damage and then aggravates ferroptosis in NP cells.

### 3.3 G3BP1 maintains the lysophagy activity and its dysfunction aggravates the lysosomal damage

**FIGURE 3:**
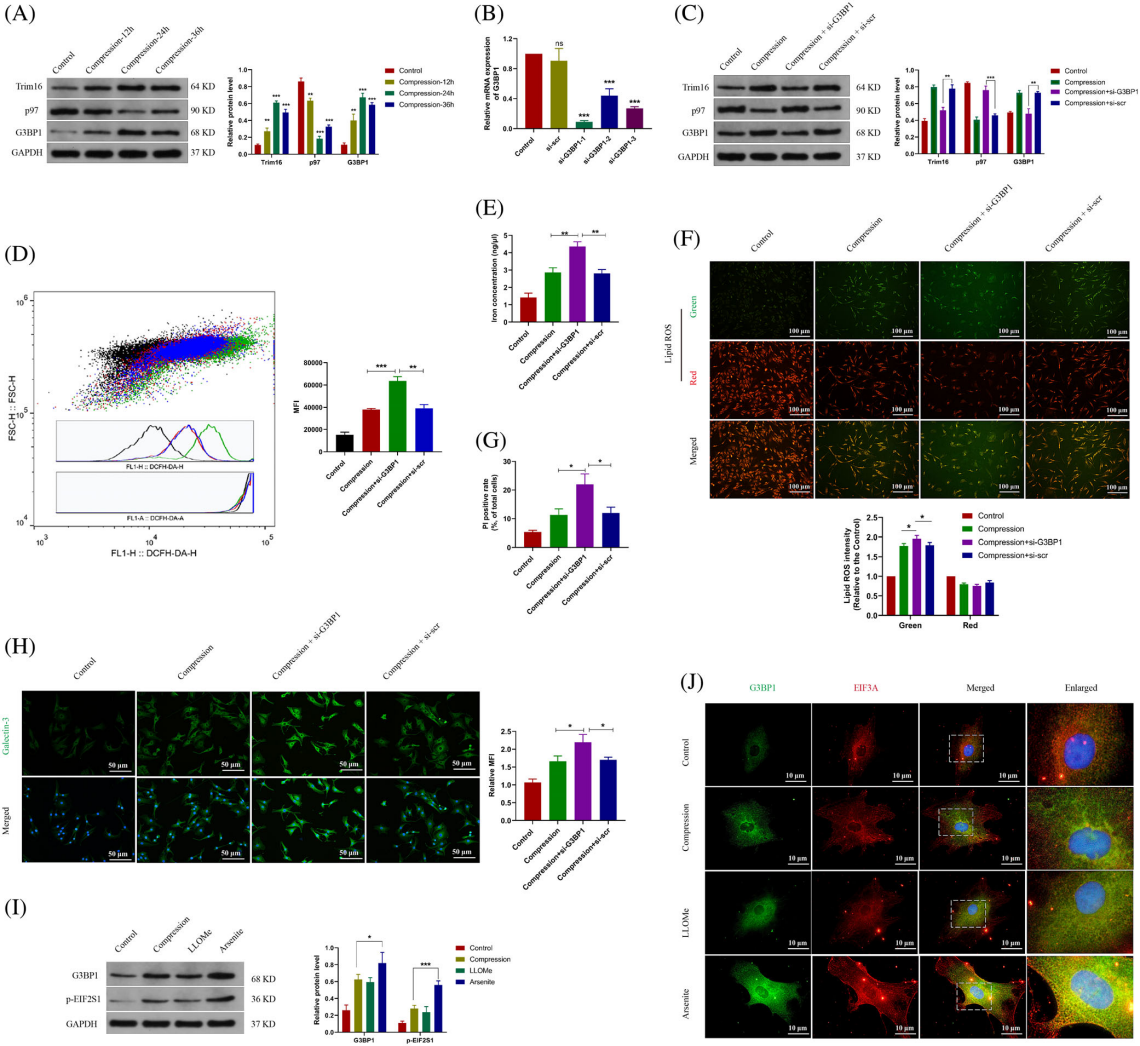
G3BP1 regulated the lysophagy and maintained lysosome function. (A) NP cells were treated with the compression pressure for 0, 12, 24 or 36 h. Western blot analysis and quantitative protein levels of Trim16, p97 and G3BP1 were shown. (B) Relative mRNA of G3BP1 in NP cells treated with G3BP1 siRNAs (si‐G3BP1). (C) NP cells were treated with si‐G3BP1 or scrambled siRNA (si‐scr). Western blot analysis and quantitative protein levels of Trim16, p97 and G3BP1 were shown. (D) Flow‐cytometry of DCFH‐DA and the calculated MFI were shown. (E) The concentration of cellular free iron was measured. (F) Representative images of C11‐BODIPY staining in NP cells and the calculated relative MFI. (G) The quantitative PI‐positive cell rate of NP cells. (H) Representative immunofluorescence images of Galectin‐3 and the calculated relative MFI. (I) NP cells were treated with the compression pressure or LLOMe or Arsenite. Western blot analysis and quantitative protein levels of G3BP1 and phosphorylated EIF2S1 (p‐EIF2S1) were shown. (J) Representative immunofluorescence images of G3BP1 (Green) and EIF3A (red). MFI, mean fluorescence intensity; NP, nucleus pulposus; PI, propidium iodide. Data were presented as mean ± SD (*n* = 3). *p < 0.05, **p < 0.01 and ***p < 0.001.

Lysophagy is one of the selective autophagy to remove cellular damaged lysosomes. Trim16, an E3 ubiquitin‐protein ligase, plays a role in lysophagy initiation and recruits the adapter valosin‐containing protein (VCP/p97) to induce lysophagy.^34^ We observed that the levels of Trim16 and p97 altered during the compression treatment (Figure 3A). It seemed that prolonged pressure impaired the lysophagy activity, as well as the level of G3BP1. To further unravel the role of G3BP1 in lysophagy, the siRNAs were used to knockdown the expression of G3BP1 (Figure 3B). It was indicated that G3BP1 knockdown decreased the expression of Trim16 and induced the accumulation of p97 (Figure 3C). Meanwhile, decreased activity of lysophagy was associated with the elevated ROS level (Figure 3D). The levels of cellular free iron and lipid peroxidation were increased in the G3BP1 knockdown group (Figure 3E,F). It was indicated the G3BP1 dysfunction aggravates the cell ferroptosis (Figure 3G). We found that G3BP1 dysfunction induces the accumulation of Galectin‐3 and lysosomal damage (Figure 3H), which is essential to exacerbate ferroptosis upon the compression pressure.

G3BP1 is considered as the central switch to assemble stress granules (SGs).^21^ Herein, we intended to determine whether G3BP1 is involved in SG formation under the compression pressure. Arsenite is a common drug used to induce SG assembly. It was indicated that arsenite as the positive control induced the higher levels of the SG marker phosphorylated translation initiation factor 2 alpha (EIF2A/EIF2S1) and G3BP1 compared with the compression and LLOMe group (Figure 3I). Immunofluorescence of G3BP1 and translation initiation factor 3 alpha (EIF3A) showed that G3BP1 has a significant co‐localization with EIF3A in the Arsenite group, indicating the formation of G3BP1‐associated SG. It revealed that the formation of G3BP1‐associated SG in the compression group was not as obvious than in the Arsenite group (Figure 3J). These results demonstrated that G3BP1 more likely acts as the modulator of lysosomal activity but also the SG component under the compression pressure.

### 3.4 G3BP1 coordinates the lysophagy activity through modulating the mTOR signalling

**FIGURE 4:**
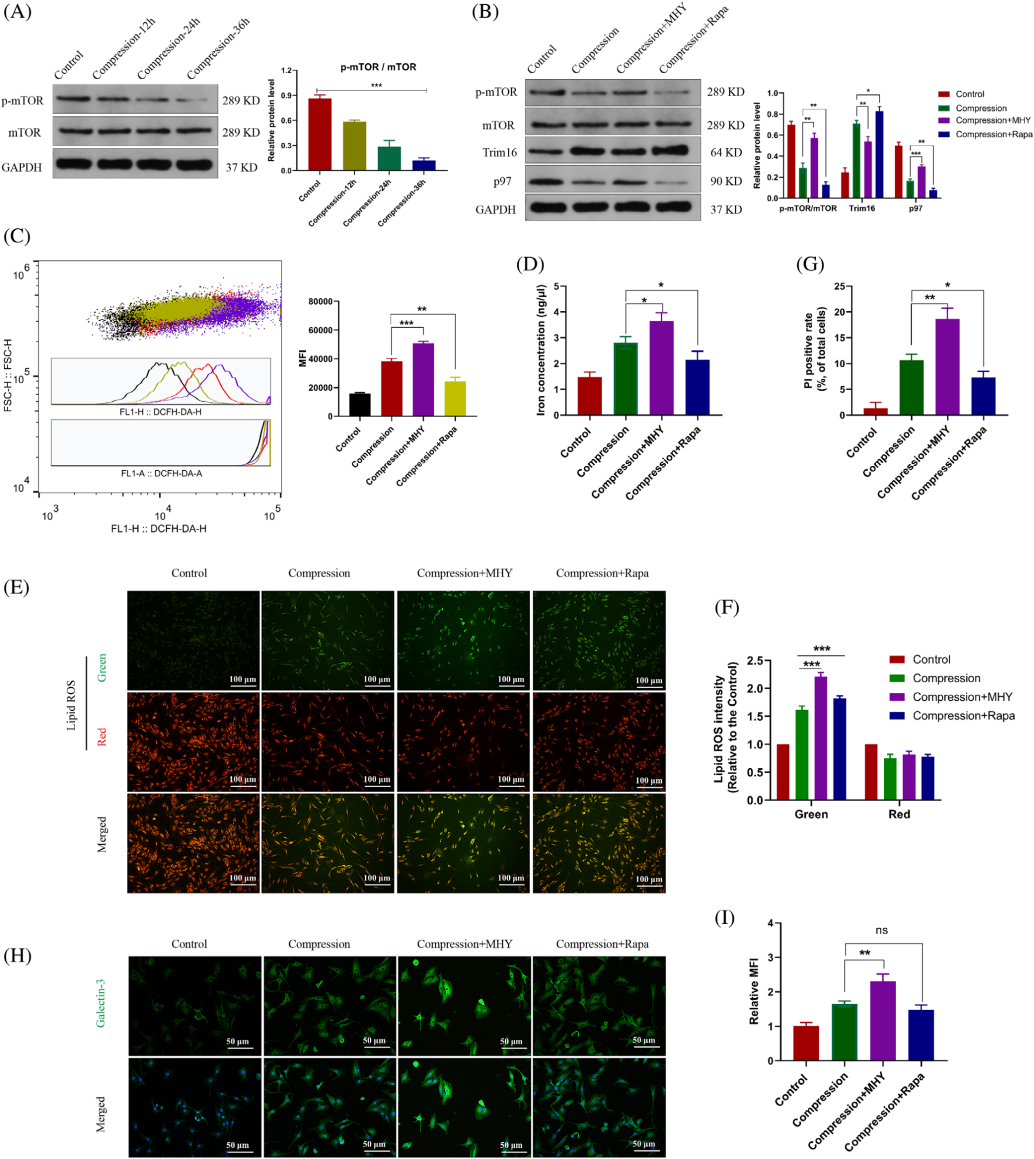
G3BP1 modulated the lysophagy and the ferroptosis outcome via mTOR signalling. (A) NP cells were treated with the compression pressure for 0, 12, 24 or 36 h. Western blot analysis and quantitative protein levels of p‐mTOR and mTOR were shown. (B) NP cells were treated with MHY1485 (MHY) or rapamycin (Rapa). Western blot analysis and quantitative protein levels of p‐mTOR, mTOR, Trim16 and p97. (C) Flow‐cytometry of DCFH‐DA and the calculated MFI were shown. (D) The concentration of cellular free iron was measured. (E, F) Representative images of C11‐BODIPY staining in NP cells (E) and the calculated relative MFI (F). (G) The quantitative PI‐positive cell rate of NP cells. (H, I) Representative immunofluorescence images of Galectin‐3 (H) and the calculated relative MFI (I). Data were presented as mean ± SD (*n* = 3). MFI, mean fluorescence intensity; NP, nucleus pulposus; PI, propidium iodide. *p < 0.05, **p < 0.01 and ***p < 0.001, and *ns* for no significant difference.

Mammalian target of rapamycin (mTOR) signalling controls lysosomal metabolic pathways.^35^ We suspect that G3BP1 regulates lysosomal activity via the mTOR signalling. It was indicated the level of phosphorylated mTOR was decreased during the compression pressure, revealing the inhibition of mTOR signalling (Figure 4A). NP cells were treated with the mTOR signalling activator MHY1485 or the specific mTOR inhibitor rapamycin. We found that the lysophagy activity was simultaneously inhibited by the mTOR activator and activated by the mTOR inhibitor (Figure 4B). Furthermore, MHY1485 treatment also induced the elevated ROS level in NP cells (Figure 4C). The concentration of cellular free iron and the lipid ROS level were increased in the mTOR‐activating group and decreased in the mTOR‐inhibiting group (Figure 4D–F). The cell death rate was significantly increased in the MHY1485 group while decreased in the rapamycin group (Figure 4G). It was shown that activation of mTOR signalling aggravated the cell ferroptosis, which was similar as the effects of G3BP1 knockdown. Besides, MHY1485 treatment also promoted while rapamycin treatment ameliorated the lysosomal damage (Figure 4H,I). These results demonstrated that G3BP1 regulates the lysophagy and cell ferroptosis via the mTOR signalling.

### 3.5 G3BP1/TSC2 complex inactivates mTOR signalling to induce lysophagy in NP cells

**FIGURE 5:**
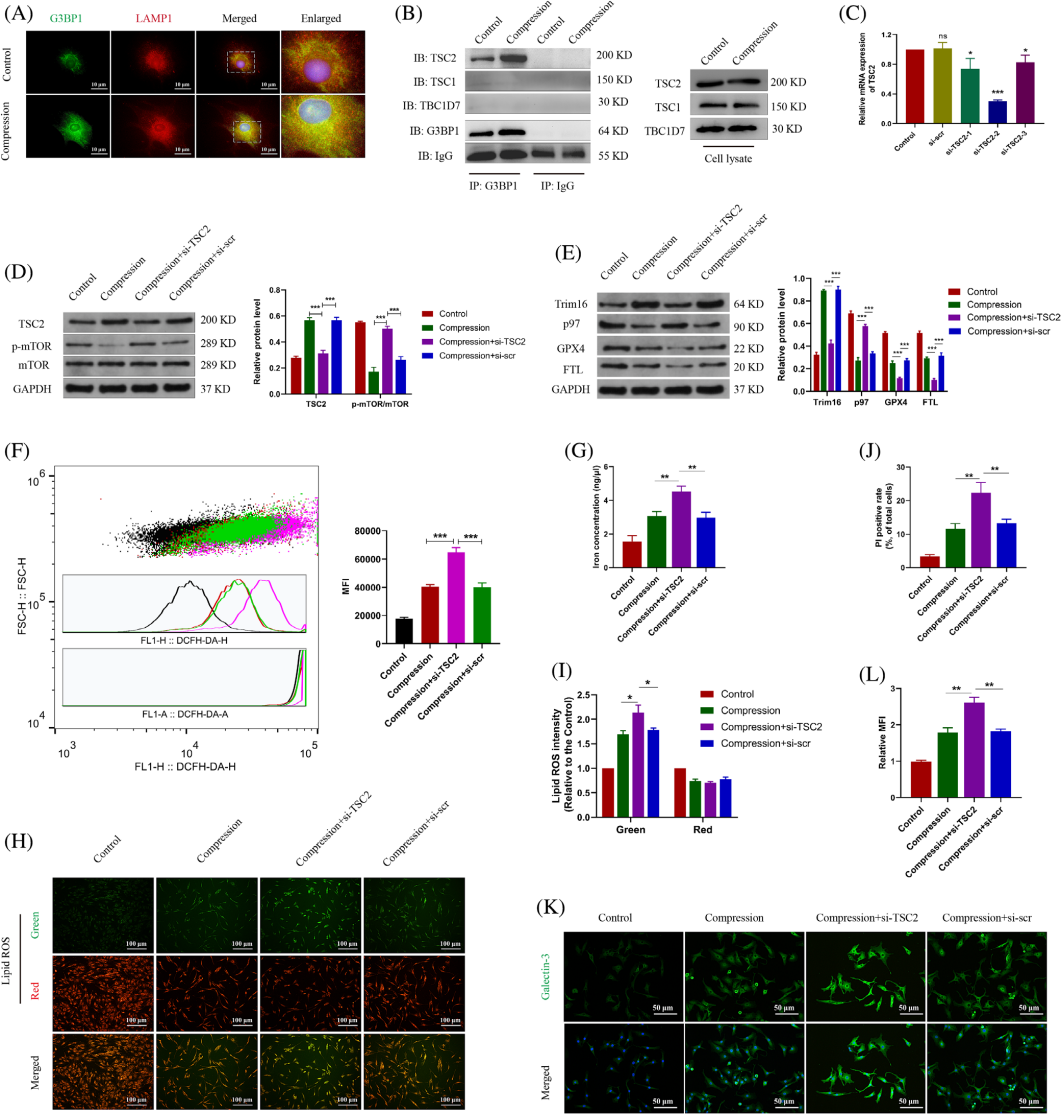
G3BP1/TSC2 complex regulated the mTOR signalling and the lysophagy activity. (A) Representative immunofluorescence images of G3BP1 (Green) and LAMP1 (red). (B) Immunoprecipitation of G3BP1 and immunoblotting of TSC1, TSC2 and TBC1D7. (C) Relative mRNA of TSC2 in NP cells treated with TSC2 siRNAs (si‐TSC2). (D) NP cells were treated with si‐TSC2 or si‐scr. Western blot analysis and quantitative protein levels of TSC2, p‐mTOR and mTOR. (E) Western blot analysis and quantitative protein levels of Trim16, p97, GPX4 and FTL. (F) Flow‐cytometry of DCFH‐DA and the calculated MFI. (G) The concentration of cellular free iron was measured. (H, I) Representative images of C11‐BODIPY staining (H) and the calculated relative MFI (I). (J) The quantitative PI‐positive cell rate of NP cells. (K, L) Representative immunofluorescence images of Galectin‐3 (K) and the calculated relative MFI (L). MFI, mean fluorescence intensity; NP, nucleus pulposus; PI, propidium iodide. Data were presented as mean ± SD (*n* = 3). *p < 0.05 and **p < 0.01.

To further investigate the role of G3BP1 in the mTOR signalling, we intend to unravel the binding of G3BP1 with mTOR complex. We observed that G3BP1 co‐localized with LAMP1, which indicates the lysosomal localization of G3BP1 (Figure 5A). The immunoprecipitation analysis showed that G3BP1 binds to mTOR subunit TSC2, not TSC1 or TBC1D7 (Figure 5B). It seemed that more level of G3BP1 co‐immunoprecipitated with TSC2 in NP cells under the compression pressure. To verify the role of TSC2 in mTOR complex, the siRNAs were utilized to knockdown the TSC2 expression (Figure 5C). It was indicated that knockdown of TSC2 induced the elevated level of phosphorylated mTOR (Figure 5D). Besides, TSC2 knockdown significantly inhibited the activation of lysophagy and decreased the level of ferroptosis‐associated protective proteins (Figure 5E). It seemed that dysfunction of G3BP1/TSC2 complex induced the inactivation of lysophagy thereby aggravated the cell death. In fact, TSC2 inhibition increased cellular ROS level and free iron concentration (Figure 5F,G). The lipid ROS level was also significantly increased in the TSC2 knockdown group (Figure 5H,I). The results of PI‐positive cell rate indicated that the level of ferroptosis was elevated after the TSC2 inhibition (Figure 5J). Besides, TSC2 knockdown also induced the increased expression level of Galectin‐3, which indicates the aggravation of lysosomal damage (Figure 5K,L). These results illustrated that G3BP1/TSC2 complex plays a role in mTOR signalling to regulate lysophagy and ferroptosis of NP cells.

### 3.6 Knockdown of G3BP1 accelerates the progression of IDD in vivo

**FIGURE 6:**
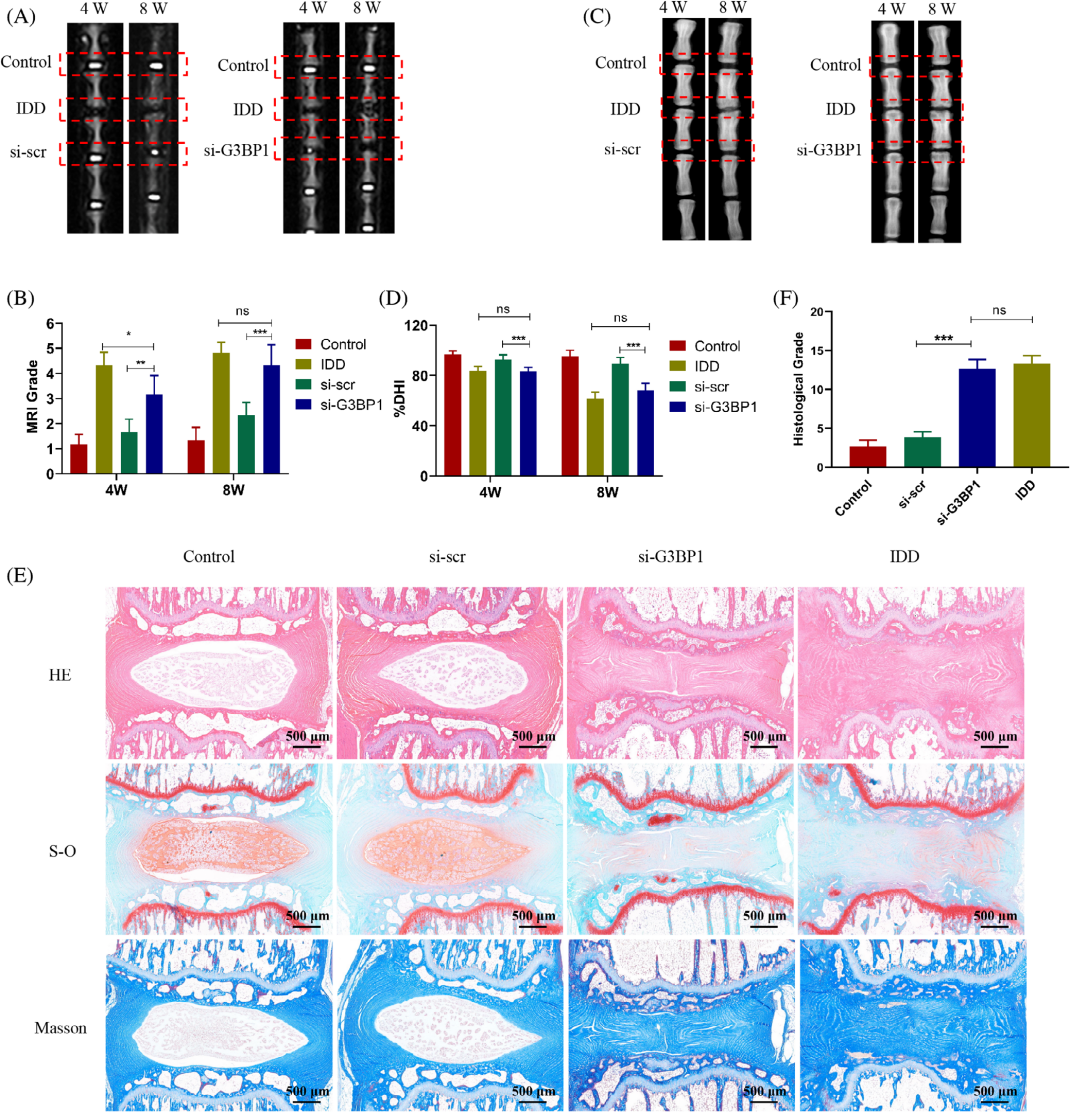
G3BP1 knockdown accelerated the progression of IDD in vivo. (A) The rat disc was injected with si‐G3BP1 or si‐scr. The representative MRI images of discs at 4 weeks and 8 weeks postoperative in different groups. (B) The MRI grade was evaluated based on the MRI images. (C) The representative X‐ray images of discs at 4 and 8 weeks postoperative in different groups. (D) Change of DHI (% DHI) was calculated based on the X‐ray images. (E) Representative images of histological staining including HE, Safranin‐O (S‐O) and Masson of discs in different groups. (F) Histological grade was evaluated based on the histological staining results. DHI, disc height index; HE, haematoxylin and eosin; MRI, magnetic resonance imaging. Data were presented as mean ± SD (*n* = 6). *p < 0.05, **p < 0.01 and ***p < 0.001, and *ns* for no significant difference.

To assess the effect of G3BP1 on IDD, a disc degeneration model is established by needle puncture of the rat tail. The siRNAs targeted for G3BP1 were injected into the disc and the degenerative degree of rat disc was evaluated. The degenerated disc is characterized by loss of water content and disc height, and decrease of resident cells. MRI images revealed that G3BP1 knockdown promoted the water content loss in discs and increased the MRI grade (Figure 6A,B). X‐ray images showed that G3BP1 knockdown significantly decreased the disc height, which was similar as the IDD group (Figure 6C,D). Besides, the histological staining results indicated that the NP area was diminished both in the IDD and the G3BP1 inhibition group (Figure 6E). The G3BP1 knockdown also promoted the tissue fibrosis and increased the histological grade of the disc (Figure 6F). These data showed that the knockdown of G3BP1 promotes the progression of IDD in vivo.

### 3.7 Inactivating the mTOR signalling retards the progression of IDD in vivo

**FIGURE 7:**
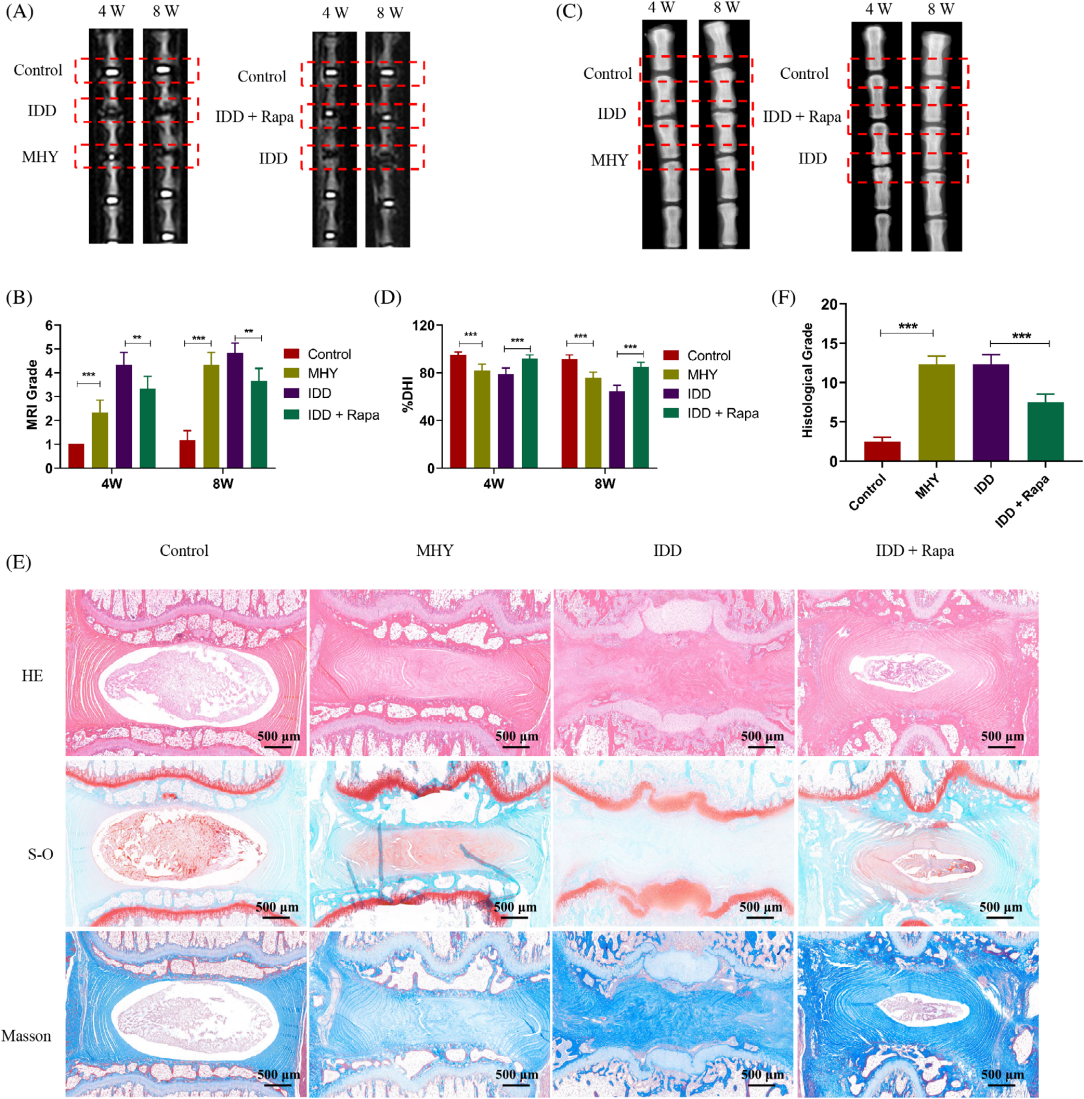
Regulation of mTOR signalling affected the progression of IDD in vivo. (A) The rat disc was injected with MHY1485 or rapamycin. Representative MRI images of discs at 4 weeks and 8 weeks postoperative in different groups. (B) The MRI grade was evaluated based on the MRI images. (C) The representative X‐ray images of discs at 4 and 8 weeks postoperative in different groups. (D) The variation of DHI was calculated based on the X‐ray images. (E) Representative images of histological staining including HE, S‐O and Masson of discs in different groups. (F) Histological grade was evaluated based on the histological staining. DHI, disc height index; HE, haematoxylin and eosin; IDD, intervertebral disc degeneration; MRI, magnetic resonance imaging. Data were presented as mean ± SD (*n* = 6). *p < 0.05, **p < 0.01 and ***p < 0.001.

To gain a further understanding of G3BP1/mTOR signalling during the progression of IDD, the drugs targeted for mTOR signalling were used in the disc degeneration model. It was indicated that mTOR activator MHY1485 increased the MRI grade of discs, which was same as the IDD group (Figure 7A,B). Besides, disc height was decreased significantly in the MHY1485 group compared with the control group (Figure 7C,D). However, mTOR inhibitor rapamycin could partly ameliorate the loss of water content and disc height during the progression of IDD. Compared with the IDD group, the MRI grade was lower and the DHI was higher in the rapamycin group. The histological staining results also revealed that the NP area was replaced by fibrous tissues in the MHY1485 group and the IDD group (Figure 7E). Compared with the MHY1485 and the IDD group, the degenerative grade was lower in the rapamycin group, indicating a less significant degenerative profile of the disc (Figure 7F). These results demonstrated that the G3BP1/mTOR signalling is involved in the progression of IDD in vivo and it may provide potential targets for IDD treatment.

## 4 DISCUSSION

**FIGURE 8:**
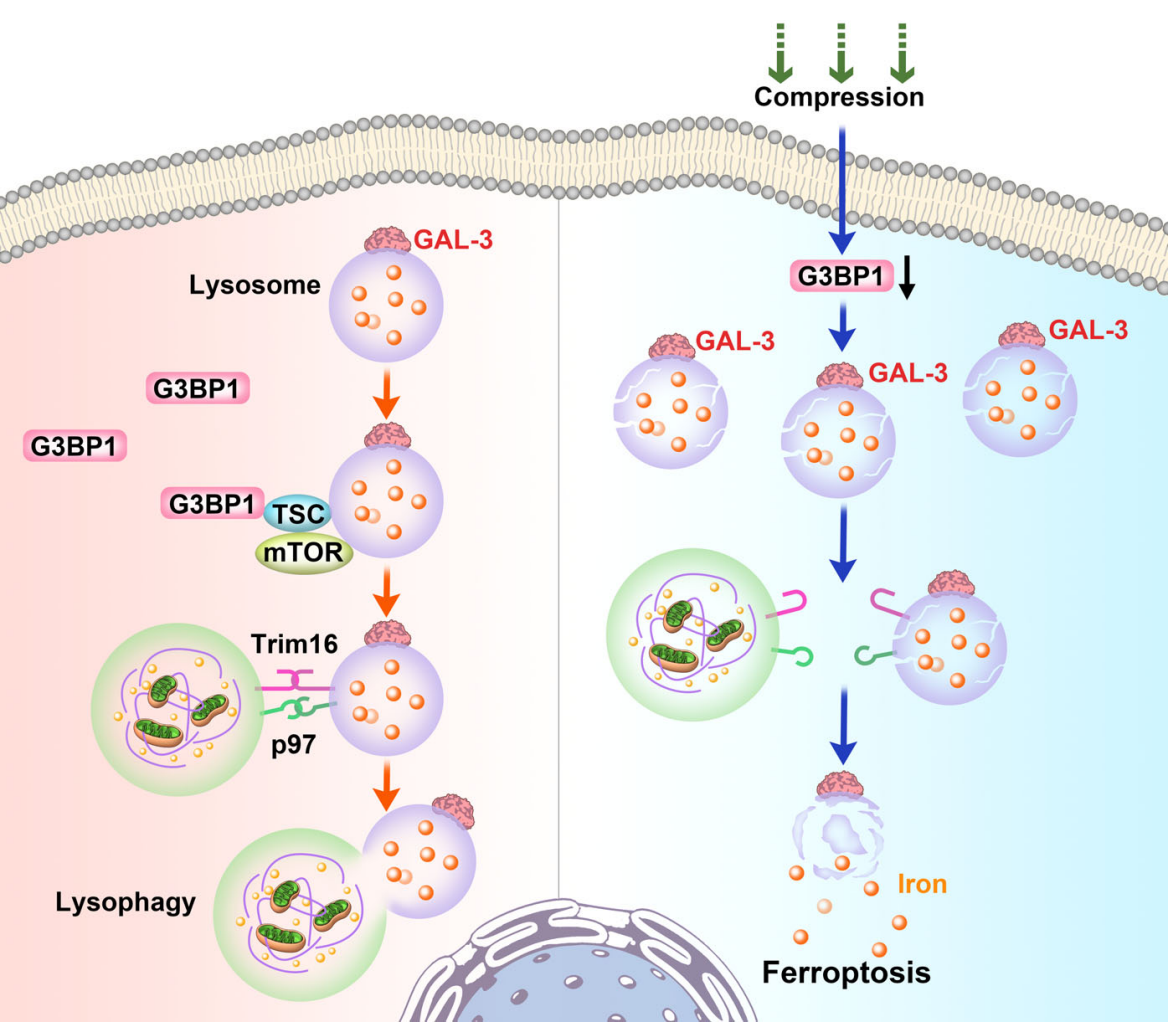
Schematic model revealing the protective role of G3BP1 in compression‐induced lysosomal damage and cell ferroptosis. G3BP1 coordinates TSC/mTOR complex to initiate lysophagy for removing injury lysosomes. Under the compression pressure, G3BP1 dysfunction promotes the inactivation of lysophagy. Accumulation of damaged lysosomes induces the elevated levels of free iron and lipid peroxidation, resulting in cell ferroptosis.

Growing evidence has revealed that cell ferroptosis and lysosome damage make an effect on the progression of IDD.^20^, ^36^, ^37^ Lysophagy regulates the quantity and quality of lysosome, which maintains intracellular homeostasis under stress conditions.^38^ However, the regulators of lysophagy and its relationship between ferroptosis remain unclear. Here, we detected the expression of the lysosomal damage marker Galectin‐3 and a potential regulator G3BP1, and analysed their relationship with the IDD degree of human disc tissues. Insufficient activity of lysophagy could not protect NP cell against ferroptosis upon compression stress. Besides, dysfunction of G3BP1 induced the inactivation of lysophagy in compression‐treated NP cells, resulting to an elevated level of cell ferroptosis (Figure 8). Furthermore, we found that G3BP1 regulates lysosome activity by binding to TSC2 and activating the mTOR signalling pathway. The protective roles of G3BP1 and mTOR were testified in the in vivo disc degeneration model, which may provide potential targets for IDD treatment.

IVD is the connecting part that localizes between spinal vertebrae, which serves for spine stabilization and maintains proper intervertebral space height.^39^ DDDs induced by IDD are characterized by diverse degenerative pathological changes, such as irregular cell death, cell senescence or ECM degradation.^31^, ^40^ Growing evidence has shown that diverse forms of programmed cell death are involved in the progression of IDD.^41^, ^42^ Among these, ferroptosis is the newly discovered type and its role in IDD is without adequate understanding. According to the single‐cell RNA sequencing results in a recent study, ferroptosis‐related genes were high‐expressly found in degenerative human and rat tissues.^37^ Our previous study also indicated that restore of the Ferroportin function could ameliorate intracellular iron overload and protect against NP cell ferroptosis.^18^ In this case, interventions on ferroptosis may provide new therapeutic targets for IDD therapy.

Lysosomes as the cellular catabolic hub are responsible for the degradation of proteins, aggregates or organelles.^6^ Lysosomal quality control is essential for cellular homeostasis. When irreversible lysosomal damage happens, lysophagy is initiated to clear damaged lysosomes to avoid the leakage of harmful substances and further activate the lysosome regeneration.^9^ Therefore, proper lysophagy activity may be beneficial for maintaining disc cell microenvironment. Previous studies have investigated the role of lysosomal degradation during autophagy, mainly macroautophagy.^43^ However, the selective autophagy form, lysophagy gains not enough attention. Several researches have revealed that Cathepsin B mainly released from damaged lysosomes is an important initiator and mediator of ferroptosis.^44^, ^45^ Besides, autophagy activation induces the degradation of ferritin and then releases free iron to result in pyroptosis, which is also called ferritinophagy.^46^, ^47^ Indeed, lysosomes are an important player during the process of ferroptosis.^48^ In the present study, we also found that lysophagy activity was impaired upon the compression loading and consequently the cellular iron levels were increased. Activation of lysophagy protected NP cell against ferroptosis through removing the damaged lysosomes to decrease the cellular iron level.

G3BP1, a well‐known stress‐associated protein, is related to several cellular processes.^21^, ^22^, ^24^ The function of G3BP1 is not limited to the formation of stress granules. Recent study has indicated the role of G3BP1 in lysosomal signalling.^25^ In our study, we further revealed that intervention on G3BP1 decreased the lysophagy activity and aggravated the lysosomal damage. G3BP1 may be an important player to keep the lysosomal homeostasis. Besides, diverse roles of G3BP1 in selective autophagy have been implicated recently, including ribophagy, mitophagy and autophagy for Tau degradation.^49^, ^50^, ^51^ The lysosome may be the central link between G3BP1 and autophagy. More importantly, we found that G3BP1 decreased in degenerative disc tissues and knockdown of G3BP1 significantly promoted the ferroptosis in NP cells. These results supported the protective role of G3BP1 during the progression of IDD. Treatments based on restoring G3BP1 function are not only beneficial for maintaining lysosomal homeostasis, but could provide a therapeutic target for IDD.

The TSC‐mTOR signalling is associated with autophagy activation, cell growth and inflammatory response.^52^, ^53^ Previous study has indicated that pharmacological inhibition of mTORC1 enhances autophagy to protect NP cell against apoptosis and senescence.^54^ Moreover, mTOR activity could be regulated by the class I phosphoinositide 3‐kinase (PI3K) signalling that maintains cellular metabolism and growth.^55^ When mTORC1 is activated and recruited to the lysosome, it interacts with lysosomal surface receptor and regulates the lysosomal activity.^56^ During this process, G3BP1 may be an important mediator of mTORC1 activity. We found that G3BP1 could bind to the TSC‐mTORC1 complex and inhibit mTORC1 activity, which is consistent with the previous study.^25^ In this way, G3BP1 modulates the lysophagy activity via TSC‐mTOR signalling and regulates the lysosomal homeostasis. Therefore, it is reasonable to assume that intervention of G3BP1 expression in degenerated NP cells may restore lysophagy and reduce the accumulation of damaged lysosomes to alter the cell fate.

Our study first reveals the role of G3BP1 in IDD and offers new sights into the relationship between lysophagy and ferroptosis. However, there still have some limitations in our present study. We only measure the activity of lysophagy by detecting the expression of marker proteins during a relatively short period in NP cells. For further understanding, it is of great value to monitor lysophagy for a longer period of time. Besides, we obtain our results based on the complication of static pressure loading in NP cells. Strictly, dynamic pressure with varying pressure conditions is more suitable for modelling, which is closer to the physiological state of NP cells in disc. Finally, the role of G3BP1 in the lysosomal function may be limited in our study. The underlying mechanism of G3BP1 regulating the TSC‐mTOR signalling pathway still remains unclear, including the unknown modifications that control their binding or activation.

## 5 CONCLUSION

In summary, our study investigates the relationship between the lysophagy, lysosomal damage and cell ferroptosis. G3BP1 as the core tether coordinates the lysophagy activity to remove injured lysosomes, which inhibits the overload of cellular free iron. Under the compression pressure, it seems that G3BP1 mainly resides at lysosomes to regulate lysosomal function via the TSC2/mTOR signalling and not forms stress granules. Our finding illustrates the role of G3BP1 and mTOR signalling in NP cell ferroptosis and may provide potential therapeutic targets for IDD.

## AUTHOR CONTRIBUTIONS

**Shuai Li:** Conceptualization, experimental operation, manuscript writing. **Zhiwei Liao:** Conceptualization, experimental operation, manuscript writing. **Huipeng Yin:** Data collection; Ouyang Liu, data collection. **Wenbin Hua:** Data collection. **Xinghuo Wu:** Data collection. **Yukun Zhang:** Data collection. **Yong Gao:** Conceptualization, data curation, funding acquisition, supervision. **Cao Yang:** Conceptualization, data curation, funding acquisition, supervision.

## CONFLICT OF INTEREST

The authors declare no conflict of interest.

## Data Availability

All data generated during this study are included in this article
